# Exploring the gap: attitudes vs. purchasing behavior in sustainable outdoor sports apparel

**DOI:** 10.3389/fspor.2026.1736169

**Published:** 2026-02-09

**Authors:** Josef Voráček, Daniel Opelík, Jana Veselá, Petra Hospodková

**Affiliations:** 1 Faculty of Physical Education and Sport, Department of Sport Management, Charles University, Prague, Czechia; 2 Faculty of Biomedical Engineering, Department of Biomedical Technology, Czech Technical University in Prague, Kladno, Czechia

**Keywords:** decision-making, sports marketing, sustainability, sustainable apparel, theory of planned behavior

## Abstract

**Objective:**

The objective of this study is to explore the discrepancy between consumers’ stated attitudes toward sustainability and their actual purchasing-related evaluations within the context of outdoor sports apparel. Using the Theory of Planned Behavior (TPB) as an interpretive framework, the study examines how key TPB-related factors manifest in descriptive patterns of consumer responses. The study employs an exploratory quantitative approach to identify how these theoretically grounded factors relate to purchase probability and willingness to pay for sustainable outdoor apparel.

**Methods:**

The research employs a quantitative methodology based on electronic surveying. Data were obtained from 311 respondents who actively engage in outdoor sports and activities. The theory of planned behavior is applied for data analysis, allowing for a deeper understanding of customer preferences and specific consumer behavior.

**Results:**

The results indicate that price perception plays a crucial role, with the higher price of sustainable apparel being a significant barrier to purchasing decisions. The study further revealed that consumer awareness of sustainable attributes positively influences their willingness to purchase and accept higher prices. The findings also suggest that situational factors can significantly impact the final purchasing decision.

**Conclusion:**

The article provides recommendations for outdoor companies on how to effectively communicate the sustainable attributes of products, increase customer awareness, and mitigate the impact of perceived high prices. The study's conclusions support the idea that educational campaigns aimed at clarifying the importance of sustainability and the practical benefits of sustainable products can lead to changes in purchasing behavior and promote sustainable consumption in the outdoor industry. The study emphasizes the need to focus on communicating the practical benefits of sustainable products, such as their longevity and durability.

## Introduction

Sustainability is defined as the ability to meet present needs without compromising the ability of future generations to meet their own needs, emphasizing economic, social, and environmental dimensions. In the outdoor industry, sustainability is crucial because outdoor activities depend on the preservation of natural resources and ecosystems. Increasing consumer awareness of the environmental impacts of production and consumption (1) has led to a growing interest in sustainable practices (2, 3). However, despite positive attitudes towards sustainability, there often exists a discrepancy between these attitudes and actual purchasing behavior, known as the attitude-behavior gap.

The outdoor industry has seen an increase in the adoption of sustainable practices, with many brands incorporating recycled materials, ethical labor practices, and environmentally friendly production methods. Brands like Patagonia and Fjällräven have built their marketing strategies around sustainability, appealing to environmentally conscious consumers. Despite these efforts, the higher price of sustainable products often deters consumers from making eco-friendly purchases (4–9). This study tries to explore the factors contributing to this discrepancy and provide insights into how outdoor brands can better align consumer attitudes with purchasing behavior.

Previous research has highlighted the importance of price (10–12), awareness (13, 14), and situational factors (15, 16) in influencing sustainable purchasing behavior. Studies have shown that consumers are willing to pay a premium for sustainable products, but this willingness is often limited by their budget constraints and perceived product value. Additionally, situational factors such as the retail environment and skepticism about greenwashing can significantly impact purchasing decisions.

This study focuses on a deeper exploration of this discrepancy in the context of purchasing outdoor apparel. The research is based on the theory of planned behavior (TPB), which provides a framework for understanding the relationship between attitudes, norms, perceived behavioral control, and purchase intention (17, 18). Using the Theory of Planned Behavior (TPB) as an interpretive framework, the study examines how key TPB-related factors—such as awareness of sustainable attributes, perceived price barriers, and subjective norms—manifest in descriptive patterns of consumer responses. Rather than aiming to statistically test TPB, the study employs an exploratory quantitative approach to identify how these theoretically grounded factors relate to purchase probability and willingness to pay for sustainable outdoor apparel.

Unlike previous studies that have applied the Theory of Planned Behavior (TPB) to the domain of sustainable fashion in general (19), this paper introduces a modified TPB model that incorporates price perception, the influence of the outdoor community, and situational factors in purchasing decisions. This approach expands the traditional framework for explaining the discrepancy between declared attitudes and actual consumer behavior.

A specific contribution of this study lies in its focus on the Czech (CEE region) context, which has not yet been systematically examined in relation to TPB and sustainable consumption—particularly within the domain of sport, where existing research remains scarce (20, 21). The Czech Republic is characterized by a strong popularity of outdoor activities, coupled with a higher price sensitivity compared to Western European markets (e.g., Scandinavia, Germany). This economic-cultural framework may influence consumers’ willingness to pay a premium for sustainable products, and thus the extent of the “attitude–behavior gap.” International research often shows that in countries with a strong environmental culture, the gap between attitude and behavior tends to be smaller (5, 7, 21, 22), whereas in contexts with lower levels of environmental literacy or lower household income, this gap is more pronounced.

In this regard, the Czech case offers a relevant contrasting perspective and contributes to a deeper understanding of the influence of economic and cultural factors on sustainable consumer behavior in Central Eastern Europe (CEE region). The Czech Republic can be considered a representative example of the CEE region, given its unique position, well-developed institutional infrastructure, and cultural characteristics that reflect broader regional patterns. As highlighted by Hospodková et al. (23), Czechia serves as a suitable model for examining regional dynamics due to its active engagement in sport, and its comparability with neighboring CEE countries in terms of socio-economic conditions and consumer behavior.

## Literature review

### Sustainable outdoor products

Sustainability is a central theme of this work and must be contextualized within consumption and outdoor equipment. It is important to emphasize that although this concept is widely accepted, it remains a controversial topic with various interpretations (24–27). It is most commonly defined as the effort to meet the needs of the present generation without compromising the ability of future generations to meet their own needs. This definition emphasizes the balance between economic, social (28), and environmental aspects (3, 29–31).

In the context of consumption, it is necessary to distinguish between two perspectives on sustainability (8, 24, 32). The objectivist approach focuses on determining the maximum sustainable levels of consumption. This approach seeks to objectively determine the maximum sustainable levels of consumption and the measures that need to be taken to ensure these levels are not exceeded (24). The alternative approach emphasizes the limited nature of resources and the unevenness of their consumption, leading to the promotion of sustainable consumption that minimizes environmental impacts. This approach focuses on equitable consumption, where consumption would be socially fair and minimize environmental impacts (10).

Given the current unsustainable models of production and consumption, attention must be paid to the concept of the circular economy (CE). CE focuses on minimizing waste and transforming it into a resource, thereby reducing dependence on primary raw materials and minimizing negative environmental impacts (7).

The life cycle of a sustainable outdoor product emphasizes ethics and long-term use (33). Key aspects include (34):

Production Materials (34–36): Prioritizing environmentally friendly materials that minimize environmental impact. A significant step is the ban on PFC substances in the European Union since 2020. Both natural and synthetic materials have their advantages and disadvantages. Synthetic fibers are accessible and wrinkle-resistant but release microplastics (33). Natural fibers are antibacterial and thermally insulating.
Social Sustainability: Includes fair wages and safe working conditions for all workers in the supply chain (37–39).
Region of Production: Manufacturing in countries with strict labor standards ensures adherence to ethical norms and minimizes negative social impacts (39, 40).
Factories: It is important to establish partnerships with ethical and environmentally friendly factories that adhere to high environmental and social standards (39).
Additional Services: Offering services such as repairs and extending the product's lifespan is becoming standard and contributes to sustainability (33, 41, 42).

### Attitudes vs. Purchasing Behavior

To understand the factors influencing consumer behavior in the realm of sustainable outdoor apparel, the theory of planned behavior (TPB) is crucial. This theory, extending the theory of reasoned action, considers attitudes, subjective norms, and perceived behavioral control (18, 43, 44). Attitudes represent individual evaluations of behavior, encompassing positive or negative assessments of the given behavior (45). Subjective norms represent perceived social pressure (45). Perceived behavioral control is defined as the perception of the ease or difficulty of performing the behavior (18, 45, 46).

**Figure 1:**
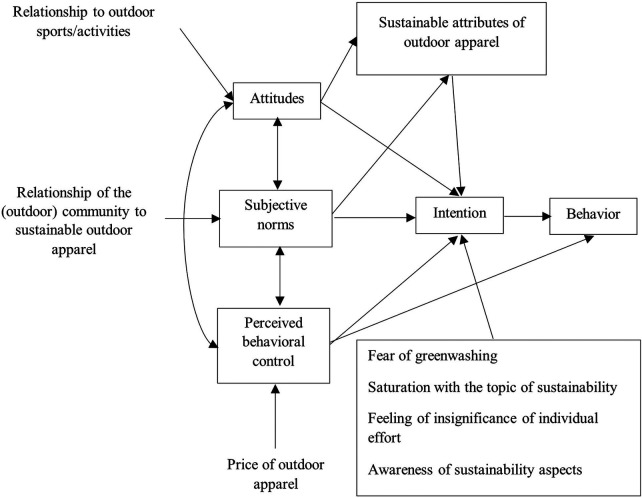
Theoretical framework of the modified schema of the theory of planned behaviour.

TPB is utilized to examine sustainable behavior in various domains, including consumption (47, 48). Despite the growing ethical consumption, the attitude-behavior gap persists (11, 13, 15, 16, 49). Studies aim to identify barriers to sustainable purchasing, such as price, convenience, and perceived quality. The priority is to find factors related to this discrepancy. The theoretical framework for this study is illustrated in Figure 1.

In the customer decision-making process, the attributes of sustainable outdoor products play a crucial role. These attributes include (9, 27, 50) the use of environmentally friendly production materials, the country (region) of production, aspects of social sustainability, the possibility of manufacturer repairs, durability, and longevity.

Key factors influencing the attitude-behavior gap include price (4, 5, 7), community attitudes towards sustainability, awareness of sustainability aspects (5, 7, 14), saturation with the topic of sustainability (5, 51, 52) and the feeling of insignificance of individual efforts (48). The high price of sustainable products is often cited as a key reason for this discrepancy. Based on the synthesis of prior studies and empirical evidence, the following research hypothesis is proposed for the present investigation:

**H1:** There is a statistically significant difference between (a) the probability of purchasing outdoor apparel with sustainable attributes and (b) the premium price consumers are willing to pay for such apparel, compared to non-sustainable alternatives.

This hypothesis is grounded in the theoretical and empirical framework established by previous research, which highlights the complex interplay between consumer attitudes, purchasing intentions, and willingness to pay in the context of sustainable consumption.

Awareness of sustainability aspects is a fundamental prerequisite for the intention to purchase sustainable products (52–54). Saturation with the topic of sustainability and the feeling of insignificance of individual efforts can lead to the rejection of purchasing such products (52). People may refuse to buy sustainably because they are tired of the constant pressure and information about the importance of sustainable behavior (48).

## Methods

The study employs quantitative research methods, specifically an electronic questionnaire distributed via social media in groups related to outdoor sport activities. The questionnaire was distributed through targeted posts in outdoor-sports social media groups (e.g., hiking communities, trail-running groups, cross-country skiing groups, climbing groups, and outdoor-gear forums). In total, nine social-media groups were involved, all of which were regionally oriented toward the Czech Republic and adjacent cross-border micro-regions. Data were collected between May and June 2024.

**Figure 2:**
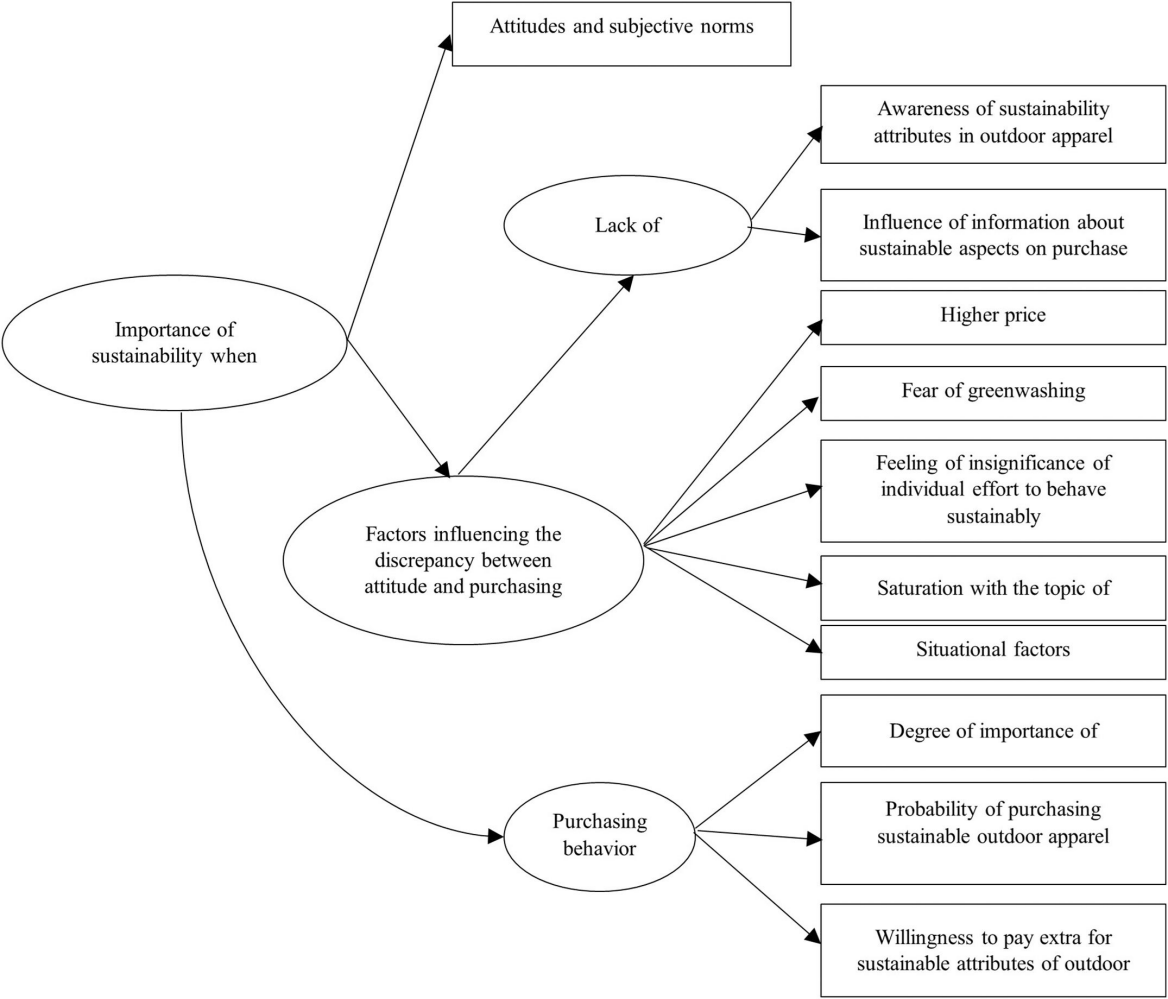
Operationalization of the construct studied.

Due to the extensive variability of products falling under the category of outdoor equipment, the research focuses on the segment of outdoor sports apparel. The questionnaire targets awareness, the importance of sustainable attributes, the influence of personal attitudes vs. subjective norms, factors influencing the attitude-behavior gap, the likelihood of purchase, and the willingness to pay a premium for sustainability. All of this is illustrated in Figure 2, which presents the operationalization of the examined issue.

### Research sample

The research sample consists of 311 respondents who engage in outdoor sports and activities. The inclusion criteria specified participation in outdoor activities and age ≥18 years. The exclusion criterion was incomplete questionnaire submissions. In total, six respondents were excluded during data cleaning. The resulting structure of the respondents is presented in Table 1.

**Table 1:** Structure of the research sample.

Criterion	Variants	Absolute size	Relative size (%)
Gender	Male	146	46.95
	Female	165	53.05
	Total	311	100
Age	18–19	28	9.00
	20–29	101	32.48
	30–39	67	21.54
	40–49	75	24.12
	50–59	33	10.61
	60 and older	7	2.25
	Total	311	100
Education	Secondary school	119	38.26
	University	192	61.74
	Total	311	100

### Quantitative data collection

In the questionnaire, most questions utilized scaling, specifically using star ratings to capture the importance of individual attributes and factors. Additionally, closed-ended questions with a single possible answer were used to record demographic characteristics, attitudes, and purchasing behavior (likelihood of purchasing sustainable outdoor apparel and willingness to pay a premium for sustainability attributes). The questionnaire also included four open-ended questions, one of which served as a filter question to potentially exclude respondents who do not engage in any outdoor sports or activities. The questionnaire employed several response scales: a 10-point star-rating scale (1 = “least important”, 10 = “most important”), an 11-point scale (−5 to +5), a 0%–100% probability scale for purchase likelihood, and willingness-to-pay items presented in percentage-based categories. The variables measuring purchase probability and willingness to pay a premium were recoded into a numeric 1–10 scale based on their respective percentage categories.

The questionnaire comprised both closed- and open-ended items. Key constructs were operationalized as follows:

Awareness of sustainable attributes: Assessed through an open-ended item (“Please describe what sustainability means to you in the context of outdoor apparel”). Responses were subsequently coded according to product life-cycle phases.
Importance of attributes: Measured using five items rated on a 1–10 scale (1 = least important, 10 = most important), for example: “How important is durability and longevity when purchasing outdoor apparel?”
Purchase probability: Respondents indicated the likelihood of purchasing sustainable outdoor apparel using a 0%–100% probability scale.
Willingness to pay a premium: Measured via items asking respondents to specify the maximum acceptable percentage price increase for sustainable attributes, expressed on a 0%–100% scale.
Attitudes vs. subjective norms: Assessed using an 11-point semantic differential scale (−5 = solely my own attitude, 0 = both equally, +5 = solely the attitude of close persons/community).

The questionnaire further included a custom text summarizing the sustainable attributes of the product throughout its life cycle. This summary provided respondents with a better understanding of the sustainable aspects of outdoor apparel for the subsequent questions. The custom text was intentionally inserted into the questionnaire only after respondents were asked about their perception of sustainability in the context of outdoor apparel. The key sections of the questionnaire are provided in Appendix A. These represent an English translation of the original instrument, which was administered in the native language of the research team.

Prior to the main data collection, the questionnaire was pilot-tested on a sample of 20 respondents from the target group to ensure the clarity of the items and the appropriateness of the scales used. The distinction between single-item and multi-item constructs is clearly presented in the operationalization overview in Figure 2 and further detailed in Appendix A. Based on the feedback received, several questions — particularly those related to attitudes toward sustainability — were revised for improved wording. The attitude items were revised following the pilot study, resulting in improved clarity and the removal of ambiguous wording. Content validity of the instrument was ensured through expert evaluation by three specialists in the fields of sports management and environmental marketing, who assessed the relevance of individual items in relation to the theoretical framework of the study.

### Data analysis

The data analysis was conducted using statistical descriptive methods, including the calculation of absolute and relative frequencies, means, standard deviations, and skewness. In the analysis, the open-ended responses were coded using a deductive coding approach based on established product life-cycle phases. Two independent coders categorized all responses, and percent agreement was used to assess inter-coder reliability. The percent agreement reached 93.14%, indicating a high level of consistency between coders.

To empirically test the hypothesis, an analysis of variance (ANOVA) will be employed. This statistical approach enables the assessment of whether the observed differences in purchase probability and willingness to pay a premium for sustainable attributes are statistically significant within the research sample. For the purposes of conducting the ANOVA, the 0%–100% probability scales for “Purchase probability” and “Willingness to pay a premium” were recoded into a 10-point scale (1 = 0%–9%, 2 = 10%–19%, 3 = 20%–29%, 4 = 30%–39%, 5 = 40%–49%, 6 = 50%–59%, 7 = 60%–69%, 8 = 70%–79%, 9 = 80%–89%, 10 = 90%–100%).

### Ethical considerations

This study was conducted in full compliance with the ethical standards required for academic research involving human participants. The research employed a quantitative methodology through an anonymous online survey, ensuring that no personally identifiable information was collected or stored. The study adhered strictly to the guidelines set forth by the Faculty Ethics Committee for anonymous online quantitative research. All necessary ethical principles — including voluntary participation, data protection, and confidentiality — were rigorously observed throughout the research process.

## Results

This chapter presents the detailed findings of the research obtained through quantitative data collection using an electronic questionnaire. The research focused on respondents’ awareness of sustainable attributes of outdoor apparel, the importance of these attributes in purchasing decisions, the influence of personal attitudes vs. subjective norms, the significance of factors affecting the attitude-behavior gap, the likelihood of purchasing sustainable outdoor apparel, and respondents’ willingness to pay a premium for sustainable attributes.

### Respondents’ awareness of sustainable attributes of outdoor apparel in the context of the product life cycle

Respondents’ awareness of sustainable attributes of outdoor apparel in the context of the product life cycle was assessed using an open-ended question. The responses (e.g., “environmentally friendly production”, “ecological packaging”, “no use of plastic”, “guaranteed repair by the manufacturer”, “production from recycled materials”, “fair treatment of animals”) were subsequently categorized into six groups according to the product life cycle phase: production, distribution, usage, damage, end of life, and overall lifespan. Table 2 summarizes the frequency and proportion of responses and respondents in each category.

**Table 2:** Respondent awareness of sustainability attributes within the product life cycle context.

Product life cycle	Frequency of responses	Percentage of responses	Percentage of respondents
Production	228	37.25%	73.31%
Distribution	76	12.42%	24.44%
Usage	42	6.86%	13.50%
Damage	58	9.48%	18.65%
End of life cycle	51	8.33%	16.40%
Overall lifespan	157	25.65%	50.48%

The results indicate that a relative majority of responses (37.3%) pertained to the production phase of the product. A total of 228 respondents (73.3%) associate the term sustainability in the context of outdoor apparel with aspects related to production, such as the use of recycled or organic materials and the reduction of water and energy consumption. The second most frequently mentioned category was the overall lifespan of the product, cited by 157 respondents (50.5%). Sustainability aspects related to distribution, usage, damage (e.g., repairability), and end of life (e.g., recycling) were mentioned significantly less frequently.

While the open-ended question suggests that awareness of sustainable attributes is primarily focused on production, a subsequent closed-ended question asked whether some of the information about sustainable aspects across the product life cycle was new to the respondents (this information was provided to respondents in the text between questions). Nearly half of the respondents (44.7%) indicated that this information was not new to them. This slight discrepancy between the responses to the mentioned questions can be explained by the fact that respondents may be aware of various sustainable initiatives (e.g., repair guarantees), but do not explicitly associate these with the general term “sustainability” during spontaneous recall.

### The importance of sustainability attributes when purchasing

Using 5 questions in the questionnaire, respondents rated the importance of five defined sustainability attributes when purchasing outdoor apparel on a ten-point scale (1—least important, 10—most important): durability and longevity, the possibility of manufacturer repairs, aspects of social sustainability, the country (region) of production, and the use of environmentally friendly production materials. Table 3 presents the statistical data (mean, standard deviation, skewness coefficient) for each of these attributes.

**Table 3:** The importance of sustainability attributes when purchasing.

Sustainability attributes	M	SD	Skewness
Durability and longevity	8.49	1.16	−0.54
Manufacturer repair	6.67	1.77	−0.54
Social sustainability	3.90	2.24	0.69
Country (region) of production	4.46	2.31	0.29
Environmentally friendly production materials	4.86	2.23	0.03

1—least important, 10—most important.

The results indicate that respondents attribute the highest level of importance to the attribute of product durability and longevity (mean 8.49). Following with a slightly lower but still significant mean of 6.67 is the possibility of manufacturer repairs. The other three attributes—social sustainability aspects (mean 3.90), country (region) of production (mean 4.46), and the use of environmentally friendly production materials (mean 4.86)—achieved statistically similar and significantly lower mean values. The positive skewness coefficient for these three attributes suggests that while many respondents rated them as having low importance, there is a smaller group that assigns them great significance.

### Attitudes or subjective norms

The influence of personal attitudes vs. subjective norms was examined using an 11-point scale (−5—I evaluate the sustainable attributes of the product solely based on my own attitude, 0—influenced equally by both factors, +5—I evaluate the sustainable attributes of the product solely based on the attitude of close persons/outdoor community). Respondents rated what influences their opinion on the importance of sustainable attributes of outdoor apparel more. Table 4 presents the distribution of responses for the entire research sample and for respondents who indicated that they are part of a community that considers sustainable attributes important.

**Table 4:** Attitudes vs. subjective norms.

Group of respondents	M	SD	Skewness
Entire research sample	−1.56	2.38	0.13
Respondents who are part of a community that considers sustainable attributes important	−0.48	2.22	−0.57

The influence of personal attitudes vs. subjective norms was examined using an 11-point scale (−5—I evaluate the sustainable attributes of the product solely based on my own attitude, 0—influenced equally by both factors, +5—I evaluate the sustainable attributes of the product solely based on the attitude of close persons/outdoor community).

The average value of −1.56 for the entire research sample indicates a preference for personal attitudes over subjective norms when evaluating the importance of sustainable attributes. However, the high standard deviation (2.38) points to considerable variability in the responses. For the group of respondents who are part of a community that emphasizes sustainability, the average value is −0.48, indicating a more balanced influence between personal attitudes and subjective norms, with a slight, almost negligible preference for personal attitudes.

### The discrepancy between attitude and purchasing behavior

The importance of aspects influencing the attitude-behavior gap was assessed using 5 questions, where respondents rated on a ten-point scale (1—least influential, 10—most influential) the impact of high price, concerns about greenwashing, the feeling of insignificance of individual efforts, saturation with the topic of sustainability, and situational factors in the store (staff, store atmosphere, product presentation, etc.). Table 5 summarizes the statistical characteristics for each of these aspects.

**Table 5:** The importance of aspects influencing the discrepancy between attitude and behavior.

Aspects	M	SD	Skewness
Higher price	7.11	2.28	−0.75
Feeling of insignificance of individual efforts	2.89	1.76	1.14
Saturation with the topic of sustainability	3.52	2.44	1.22
Concerns about greenwashing	4.05	2.23	0.65
Situational factors in the store	6.28	1.95	−0.64

1—least influential, 10—most influential,.

The most significant factor that respondents perceive as a barrier to purchasing sustainable outdoor apparel, despite positive attitudes, is clearly the higher price of these products (mean 7.11). Situational factors in the store emerged as the second most significant factor (mean 6.28), highlighting the importance of the sales environment and customer interaction. Concerns about greenwashing (mean 4.05) and saturation with the topic of sustainability (mean 3.52) play a less significant role in the attitude-behavior gap, although they may be relevant for certain customer segments. The feeling of insignificance of individual efforts (mean 2.89) was found to be the least significant factor examined.

These results imply that to reduce the gap between positive attitudes and purchasing behavior in the realm of sustainable outdoor apparel, it is crucial to address customers’ price sensitivity and optimize situational factors in the sales environment. Measures aimed at increasing transparency and credibility of environmental claims and sensitively communicating about sustainability can also have a positive impact, although they do not appear to be primary barriers to purchase. The feeling of insignificance of individual efforts does not seem to be a significant psychological barrier in this specific context of outdoor apparel.

### The probability of purchasing sustainable outdoor apparel and willingness to pay a premium

The probability of purchasing sustainable outdoor clothing was assessed on a scale from 0 to 100%, where respondents indicated the likelihood of purchasing such clothing. The 0%–100% probability scales for “Purchase probability” and “Willingness to pay a premium” were recoded into a 10-point scale (1 = 0%–9%, 2 = 10%–19%, 3 = 20%–29%, 4 = 30%–39%, 5 = 40%–49%, 6 = 50%–59%, 7 = 60%–69%, 8 = 70%–79%, 9 = 80%–89%, 10 = 90%–100%). The descriptive statistics of the results are presented in Table 6. The average reported probability of purchasing sustainable outdoor clothing was 7.45 (60%–69% interval). The negative skewness coefficient (−1.48) suggests that most respondents reported a higher probability of purchase.

**Table 6:** Purchase probability vs. willingness to pay—descriptive statistics.

Item	M	SD	Skewness
Purchase probability	7.45	2.09	−1.48
Willingness to pay premium price	2.59	1.51	1.57

1 = 0%–9%, 2 = 10%–19%, 3 = 20%–29%, 4 = 30%–39%, 5 = 40%–49%, 6 = 50%–59%, 7 = 60%–69%, 8 = 70%–79%, 9 = 80%–89%, 10 = 90%–100%.

Respondents’ willingness to pay extra for sustainable attributes was assessed through a question where they indicated the percentage price increase they would be willing to accept for the same type of outdoor clothing but with sustainable attributes. The average willingness to pay extra for sustainable attributes was 2.59 (10%–19% interval) of the price of a comparable product without these attributes. The standard deviation (1.51) indicates significant variability in the willingness to invest in sustainability. The positive skewness coefficient (1.57) shows that most responses were concentrated at lower premium values, although there is a smaller group of respondents willing to pay significantly more. It appears that respondents are primarily willing to pay extra for attributes related to the product's longevity, such as durability and repairability.

**Figure 3:**
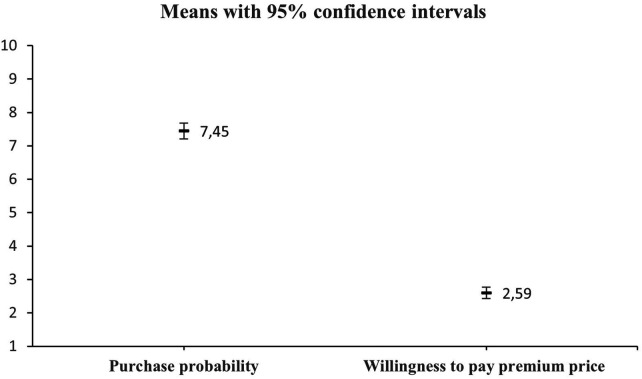
Purchase probability vs. willingness to pay (means with 95% confidence intervals). 1 = 0%–9%, 2 = 10%–19%, 3 = 20%–29%, 4 = 30%–39%, 5 = 40%–49%, 6 = 50%–59%, 7 = 60%–69%, 8 = 70%–79%, 9 = 80%–89%, 10 = 90%–100%.

**Figure 4:**
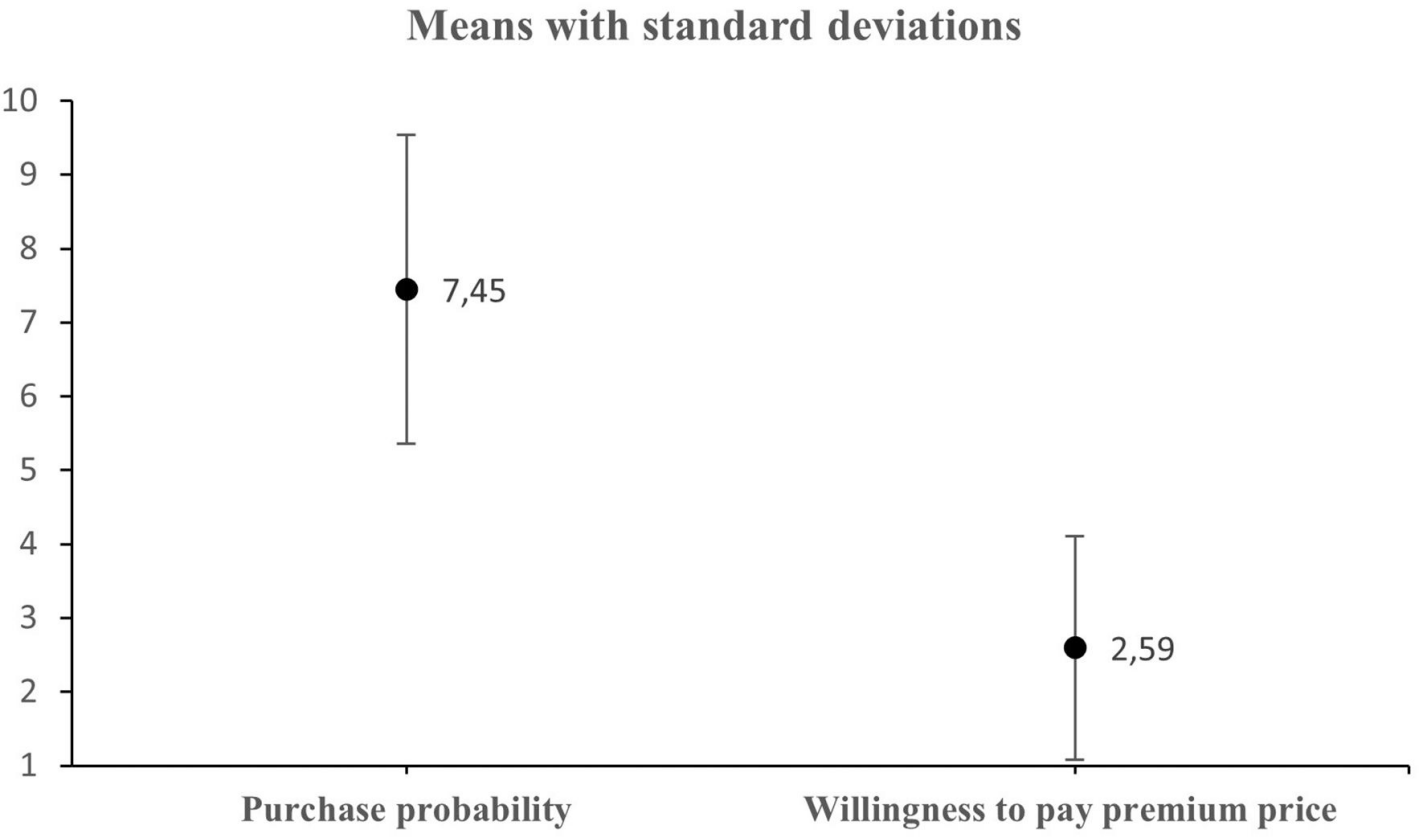
Purchase probability vs. willingness to pay (Means with standard deviations). 1 = 0%–9%, 2 = 10%–19%, 3 = 20%–29%, 4 = 30%–39%, 5 = 40%–49%, 6 = 50%–59%, 7 = 60%–69%, 8 = 70%–79%, 9 = 80%–89%, 10 = 90%–100%.

The difference between the probability of purchasing sustainable outdoor apparel and the willingness to pay a premium for such apparel was analyzed using analysis of variance (ANOVA) at a significance level of *p* = 0.05. A repeated-measures ANOVA was conducted to compare respondents’ purchase probability for sustainable apparel and their willingness to pay a price premium for such products, both assessed within subjects. The independent variable was the attribute type (purchase probability vs. willingness to pay), and the dependent variables were the corresponding rating scores. The resulting *p* = 2.59E−139 (Table 7) provides evidence of a statistically significant difference between the variables under investigation, thereby confirming the research hypothesis. The analysis yielded the following statistical results: F = 1,097.65, df = 620, *p* = 2.59E−139. Figure 3 presents the means comparison plot with 95% confidence intervals, and Figure 4 provides the means accompanied by standard deviations.

**Table 7:** ANOVA results.

Source of Variation	SS	df	MS	F	*P*-value	F crit
Between Subjects	3660.901929	1	3660.901929	1097.654635	2.5925E−139	3.856501
Within Subjects	2067.826367	620	3.335203817			
Total	5728.728296	621				

## Discussion

This study focused on exploring the discrepancy between consumer attitudes and purchasing behavior in the context of sustainable outdoor clothing. The results presented in the results section provide valuable insights into consumer awareness of sustainable attributes and their importance in purchasing decisions.

One of the key findings is the relatively limited awareness of respondents regarding sustainable attributes of outdoor clothing across the entire product lifecycle. As shown in the results (Table 2), the highest awareness is concentrated around the production phase (73.31% of respondents), while awareness of aspects related to distribution, usage, damage, and end-of-life is significantly lower. This result partially contradicts the subsequent finding that nearly half of the respondents (44.7%) stated that information about sustainable aspects across the product lifecycle was not new to them. This discrepancy suggests that consumers may have a general idea of various sustainable initiatives by brands but do not explicitly associate them with the broader concept of sustainability or specific phases of the product lifecycle. This lack of deeper understanding may be one of the barriers to bridging the gap between a positive attitude towards sustainability and actual purchasing behavior, as suggested by Yang, Song, and Tong (55), Chang and Watchravesringkan (56) and Park and Lin (57).

Regarding the importance of individual sustainable attributes, the research revealed a clear hierarchy (Table 3). Respondents attributed the highest importance to the durability and longevity of the product (average 8.49), followed by the possibility of repair by the manufacturer (average 6.67). In contrast, aspects of social sustainability (average 3.90), country (region) of production (average 4.46), and the use of environmentally friendly production materials (average 4.86) were rated as less important. This result supports the assumption that functional properties (including durability and longevity) are more important for the decision to purchase an outdoor product than the sustainability attributes themselves, which is consistent with the findings of Fuchs & Hovemann (7). However, it should be emphasized that durability and longevity can be perceived both as functional attributes and as sustainability attributes. The fact that the possibility of repair by the manufacturer ranked second in importance suggests that consumers appreciate initiatives that extend the product's lifespan and reduce the need for early replacement, which is a key principle of the circular economy (7).

The lower importance attributed to social and environmental aspects of production (country of production, environmentally friendly materials, social sustainability) may be related to the aforementioned lower awareness of these areas or the perception that these attributes do not directly affect the product's utility value for the consumer. However, the positive skewness coefficient for these three attributes (Table 3) indicates the existence of a smaller but significant group of consumers who attach high importance to these attributes. This finding is important for market segmentation and targeted communication.

The research also examined aspects influencing the discrepancy between attitude and behavior (Table 5). The most significant barrier was found to be the higher price of sustainable outdoor clothing (average 7.11). This finding is consistent with many previous studies (4, 5, 7, 9, 11, 49), which identified price as a key barrier to sustainable consumption. In contrast, the feeling of insignificance of individual effort (average 2.89) and oversaturation with the topic of sustainability (average 3.52) were found to be less significant factors. Concerns about greenwashing (average 4.05) and situational factors in the store (average 6.28) occupied a middle position. The importance of situational factors (e.g., informed staff, product presentation) underscores the significance of the retail environment in influencing the final purchasing decision.

Furthermore, the research indicated that personal attitudes play a more significant role than subjective norms when evaluating sustainable attributes of outdoor clothing, although in communities where sustainability is valued, the influence of subjective norms is stronger (Table 4). This result suggests that the primary motivation for considering sustainability is more likely to be an individual's internal conviction rather than external social pressure, which has implications for marketing communication that should focus more on consumer values and beliefs.

The study's findings generally support the application of the Theory of Planned Behavior (TPB) in the context of sustainable consumption (47, 48). Awareness of sustainable attributes can be linked to attitude towards behavior and perceived control (knowledge), price affects perceived control, and subjective norms play a role, although they did not prove to be a dominant aspect in this specific context (Figure 1). The findings on the importance of price as a barrier confirm the assumptions of the theory (4, 5, 7) that perceived difficulty (in this case, financial) can negatively affect intention and subsequent behavior.

In this study, a single research hypothesis was formulated:

**H1:** There is a statistically significant difference between (a) the probability of purchasing outdoor apparel with sustainable attributes and (b) the premium price consumers are willing to pay for such apparel, compared to non-sustainable alternatives.

This hypothesis was empirically tested using analysis of variance (ANOVA). Based on the results, the observed *p*-value (*p* = 2.59 × 10^−139^) led to the rejection of the null hypothesis (Table 7). Thus, it can be concluded that there is a statistically significant difference between the probability of purchasing (on average, respondents reported a 60%–69% higher probability) outdoor apparel with sustainable attributes and the premium price that consumers are willing to pay (on average, respondents were willing to pay only 10%–19% more) for such apparel, as compared to the same type of apparel without sustainable attributes. These findings are consistent with the results of previous studies (4, 5, 7), which have similarly identified significant disparities between purchase intentions and willingness to pay in the context of sustainable consumption.

### Implications

The findings of this study have practical implications for outdoor companies aiming to promote and sell sustainable products. Educational campaigns focused on increasing awareness of sustainable attributes across the entire product life cycle—while emphasizing not only environmental aspects but also functional benefits such as longer lifespan and repairability—appear to be crucial. Raising such awareness may enhance customers’ appreciation of sustainable attributes and help reduce perceived price barriers. In addition, the results reinforce the importance of situational factors in the retail environment, suggesting that well-informed staff, transparent communication, and an attractive presentation of sustainable products can positively shape purchasing intentions. Because concerns about greenwashing still play a secondary yet notable role, building trust and credibility through evidence-based claims and third-party certifications can further support consumer confidence.

At the managerial level, the insights show that consumers prioritize durability and repairability more than other sustainability characteristics, indicating that firms should highlight these functional benefits in marketing communication, product labelling, and point-of-sale materials. Given that the higher price of sustainable apparel emerged as the primary barrier, companies may benefit from clearly articulating the value proposition of longer-lasting products and offering price-anchoring strategies, repair services, or loyalty programs that reward sustainable choices. Retailers should also consider adjusting store environments to support decision-making—for example, by integrating sustainability information into product displays or training sales staff to address questions about materials, production, or repair options.

For scholars, the findings offer empirical indications of how TPB-related factors—attitudes, awareness, norms, and perceived behavioral control—manifest in descriptive consumer evaluations. The study contributes exploratory insights into the attitude–behavior gap in a CEE outdoor apparel context, which remains under-researched. Future research should build on these findings by employing longitudinal, experimental, or model-based statistical approaches (e.g., SEM) to test causal mechanisms and validate construct structures.

### Limitations and future research directions

Several limitations should be acknowledged. Because the study employs TPB as a theoretical frame rather than a formally tested model, the findings should be interpreted as exploratory insights into how TPB-related constructs appear in the data. The observed patterns in attitudes, awareness, and perceived barriers are therefore indicative rather than confirmatory, and they highlight areas where future TPB-based modeling (e.g., structural equation modeling or hierarchical regression) could provide stronger causal explanations.

The study relied on a non-probability convenience sample recruited through social-media groups, which may introduce self-selection bias. Participants who are more engaged in outdoor activities, more sustainability-aware, or more digitally active may have been more likely to participate. Second, the design is cross-sectional and based entirely on self-reported data, which limits causal inference and may be subject to social-desirability effects. Furthermore, the study focused specifically on sustainable outdoor apparel and did not examine other categories of outdoor equipment or general sportswear. Consequently, the findings may not fully capture consumer behavior across the broader outdoor or sports market.

This study focused on quantitative data collection using an electronic questionnaire. Future research could employ qualitative methods (e.g., in-depth interviews) to gain deeper insights into consumer decision-making processes and better understand the nuances of sustainability perceptions and factors influencing behavior. Further research could focus on specific market segments with different levels of awareness and attitudes towards sustainability, or on the impact of various types of marketing communication on bridging the gap between attitude and behavior.

## Conclusions

The aim of this study was to explore the discrepancy between consumers’ stated attitudes toward sustainability and their actual purchasing-related evaluations in the context of outdoor sports apparel. It builds on international research on the phenomenon known as the “attitude-behavior gap,” which highlights the contradiction between a positive attitude towards sustainability and the reality that consumers often do not purchase sustainably. The study contributes to the existing literature by delving deeper into this discrepancy within the specific context of the outdoor industry, which relies on the preservation of natural resources and appeals to consumers with potentially stronger connections to nature.

The originality and significance of the study lie in several key aspects. Unlike previous research, which often focused on fashion goods in general, this study specifically addresses the segment of sustainable outdoor clothing. This segment has its own specificities due to the functional requirements of the products and the potential environmental awareness of customers. The research thus fills a gap in the current knowledge, which has so far lacked a targeted discussion on sustainability from the perspective of outdoor equipment. The study also utilized the established Theory of Planned Behavior (TPB) as a framework for understanding the factors influencing purchase intention and behavior. The modified TPB model (Figure 1) incorporated specific factors relevant to the sustainable consumption of outdoor clothing, such as the relationship to outdoor activities, community attitudes towards sustainability, awareness of sustainable attributes, perceived price, concerns about greenwashing, oversaturation with the topic of sustainability, and the feeling of insignificance of individual effort.

The study provided empirical data on the awareness of Czech consumers regarding sustainable attributes of outdoor clothing across its lifecycle. It found that awareness is highest in the production phase, while it is lower in other phases (distribution, usage, damage, end-of-life). It also identified a hierarchy of the importance of sustainable attributes, with respondents attributing the highest weight to the durability and longevity of the product and the possibility of repair. These insights are crucial for targeted communication and marketing strategies of outdoor companies. The study also confirmed the significant role of price as a barrier to sustainable consumption in this segment. This finding has implications for marketing communication and brand strategies. The research emphasized the importance of situational factors in the retail environment in influencing purchasing decisions. The role of informed staff and attractive product presentation proved to be significant, highlighting the need for investment in sales staff training and optimization of sales locations.

Plans for future relevant work stem from the limitations of this study and the need for further deepening of knowledge in this area. To gain a deeper understanding of consumer decision-making processes and their perceptions of sustainability, it is recommended to conduct qualitative research, such as in-depth interviews. This method could reveal finer nuances of motivations and barriers to sustainable purchasing behavior. Future research could also focus on specific segments of the outdoor clothing market, for example, considering the type of activity performed, demographic characteristics, or level of environmental awareness. Comparing these segments could reveal additional factors influencing attitudes and behavior. It is also desirable to explore in more detail the impact of different types of marketing communication on consumer awareness of sustainable attributes and their purchase intention and behavior. Experimental studies could test the effectiveness of various communication strategies. Future research could also focus on long-term monitoring of consumer purchasing behavior exposed to various educational or marketing interventions aimed at sustainability. This would allow for the assessment of the permanence of behavioral changes. Given that this study was conducted in the Czech context, comparisons with research results in other countries could provide valuable comparative insights and reveal cultural or socio-economic specifics influencing sustainable consumption in the outdoor industry.

In conclusion, this study provides new insights into the aspects influencing the discrepancy between attitudes and purchasing behavior in the area of sustainable outdoor clothing. The identification of the key role of price and awareness, as well as the emphasis on the importance of functional attributes and situational factors, has theoretical implications for the development of consumer behavior theory in the context of sustainability and practical significance for outdoor companies seeking to communicate and sell their sustainable products more effectively. The proposed directions for future research have the potential to further deepen our understanding of this complex issue and contribute to promoting more sustainable consumption in the outdoor industry.

## Data Availability

The raw data supporting the conclusions of this article will be made available by the authors, without undue reservation.

## Appendix A. Key sections of the questionnaire.

Respondents’ awareness of sustainable attributes of outdoor apparel in the context of the product life cycle

To which outdoor sport or activity do you primarily devote your time?
What do you understand by the term “sustainability” (i.e., which characteristics, trends, initiatives, or processes) in relation to outdoor apparel?
Was any of the provided information novel to you?

The importance of sustainability attributes when purchasing

To what extent do you consider the durability and longevity of the product when purchasing outdoor apparel?
To what extent do you consider the possibility of manufacturer-provided repairs when purchasing outdoor apparel?
To what extent do you consider aspects of social sustainability (e.g., working conditions, fair wages, etc.) when purchasing outdoor apparel?
To what extent do you consider the country (region) of manufacture when purchasing outdoor apparel?
To what extent do you consider the use of environmentally friendly production materials when purchasing outdoor apparel?

Attitudes or subjective norms

On a scale, please rate the extent to which your evaluation of sustainable attributes in outdoor apparel is influenced by your personal attitude vs. the attitudes of close individuals or the outdoor community.

The discrepancy between attitude and purchasing behavior

To what extent does a higher price (compared to less expensive outdoor apparel without sustainability attributes) negatively influence your purchasing decisions?
To what extent does the feeling of the insignificance of individual efforts to behave sustainably negatively influence your purchasing decisions regarding outdoor apparel?
To what extent does oversaturation with the topic of sustainability (i.e., a negative reaction to the current frequency of use of this term) negatively influence your purchasing decisions regarding outdoor apparel?
To what extent does concern about greenwashing negatively influence your purchasing decisions regarding outdoor apparel?
To what extent do situational factors in the store (staff, store atmosphere, product presentation, etc.) influence your purchasing decisions regarding outdoor apparel?

The probability of purchasing sustainable outdoor apparel and willingness to pay a premium

What is the probability that you will purchase outdoor apparel with sustainable attributes?
What percentage above the price of outdoor apparel without sustainable attributes are you willing to pay for the same type of apparel with sustainable attributes?
